# Long Noncoding RNA ASB16-AS1 Promotes Proliferation, Migration, and Invasion in Glioma Cells

**DOI:** 10.1155/2019/5437531

**Published:** 2019-03-04

**Authors:** Delong Zhang, Huanggui Zhou, Jun Liu, Jie Mao

**Affiliations:** ^1^Department of Neurosurgery, Yijishan Hospital of Wannan Medical College, Wuhu, Anhui 241001, China; ^2^Department of Neurosurgery, Shenzhen Hospital, Southern Medical University, 1333 Xinhu Road, Shenzhen 518100, Guangdong, China

## Abstract

Glioma is a lethal, malignant intracranial tumor that becomes progressively common. It has been shown that long noncoding RNAs (lncRNAs) serve important roles in numerous diseases such as gliomas. lncRNAs can regulate the expression of targeted genes through various mechanisms. To identify a novel lncRNA that may be critical in glioma, the present study downloaded the RNA expression profiles of 171 glioma tissues and 5 normal tissues from The Cancer Genome Atlas (TCGA) database using the TCGAbiolinks package in R. Then, lncRNAs in the downloaded TCGA data were identified using the HUGO Gene Nomenclature Committee (HGNC). Based on the fragments per kilobase million value, differential expression analysis was conducted using the limma package in R. In addition, receiver operating characteristic (ROC) analysis was performed, and the area under the curve (AUC) was evaluated using the ROCR package in R. A total of 178 lncRNAs corresponding to differentially expressed genes with an AUC >0.85 were selected. Upon identifying the differential lncRNAs, ceRNA networks were constructed with these differential lncRNAs using the starbase database. From these networks, the top 10% hub genes were selected. In addition, the present study randomly selected 4 lncRNAs for quantitative polymerase chain reaction validation in tissue samples. The results revealed that lncRNA ASB16-AS1 exhibited significantly differential expression in tissue samples and was significantly associated with tumor staging and grading. Furthermore, the proliferation, invasion, and migration of U87MG and U251 glioblastoma stem-like cells (U87GS, U251GS) were significantly inhibited upon inhibition of ASB16-AS1, and the expression of key proteins in the EMT signaling pathway was affected by knocking down ASB16-AS1. Overall, the present study revealed that lncRNA ASB16-AS1 improves the proliferation, migration, and invasion of glioma cells.

## 1. Introduction

Glioma is considered the most common type of intracranial tumor. In accordance with the 2016 World Health Organization (WHO) classification [1], glioma can be classified into 4 grades, ranging from grade I to grade IV. These glioma grades can be subdivided into 2 classes: low-grade gliomas (WHO grades I and II) and high-grade gliomas (WHO grades III and IV). The vast majority of patients with glioblastoma and no history of low-grade glioma are diagnosed as primary glioblastoma [2]. Secondary glioblastomas develop from low-grade gliomas (WHO grades II-III), and these patients are usually younger and have a better prognosis. Despite the advances in surgery, radiotherapy, and chemotherapy [3], the median survival time for glioblastoma is only ~14 months under conventional treatments [4].

The major factor affecting prognosis is the surgical method employed. Tumor cells are infiltrated into adjacent tissues, which explains why certain intracranial tumors cannot be removed completely [5]. Tumor cells have an unbridled growth pattern. The heterogeneity of glioma cells makes them less susceptible to chemotherapy and radiotherapy. Thus, it is necessary to study the molecular mechanisms that affect tumor function.

Long noncoding RNAs (lncRNAs) are transcripts with a length ranging from 200 nucleotides to 100 kilobases, which have a limited protein-coding function. Increasing evidence suggests that lncRNAs modulate various functions, including cell migration, invasion, proliferation, and apoptosis. lncRNAs act as functional molecules and have been considered to serve as “RNA sponges.” lncRNAs and microRNAs (miRNAs or miRs) are capable of regulating target RNAs under ceRNA mechanisms. lncRNA H19 has been reported to act as a ceRNA for let-7 [6]. In order to identify functional lncRNAs, the present study performed a biometric analysis of the TCGA database. The results suggested that there are ~178 lncRNAs with significant differences between glioma tissues and normal tissues and an AUC >0.85. The dysregulated lncRNA-associated ceRNA network of 178 lncRNAs was built based on data exported from starBase v2.0 [7, 8].

Upon constructing the ceRNA network of differential lncRNAs, the present study randomly selected 4 differential lncRNAs from the top hub genes with 10% connectivity, and in order to verify whether they were differentially expressed in tissue samples and involved in functions of GBM stem-like cells (GSCs).

In summary, this study identified a novel lncRNA which is involved in glioma proliferation, invasion, and migration.

## 2. Materials and Methods

### 2.1. Online Database

#### 2.1.1. TCGA

The RNA sequencing (RNAseq) fragments per kilobase million (FPKM) data were downloaded from The Cancer Genome Atlas (TCGA, https://cancergenome.nih.gov/) database using the TCGAbiolinks [9] bioconductor package in R. The data consisted of 174 samples, of which 5 were normal, 13 were recurrent glioblastomas, and 156 were glioblastomas. To annotate TCGA ensembl ID, the complete HUGO Gene Nomenclature Committee (HGNC) dataset was downloaded from the HGNC database. The information provided by the HGNC dataset was employed to identify the ensembl IDs corresponding to lncRNAs. Differentially expressed lncRNAs (DElncRs) were identified using the limma [10] bioconductor package in R, with the filter criteria set to |log_2_ fold-change| >1.0 and adjusted P<0.001. Receiver operator characteristic curve (ROC) analysis was conducted using the R package ROCR.

#### 2.1.2. StarBase v2.0

StarBase was developed to predict protein-RNA and miRNA-target interactions. The present study built a predicted ceRNA network about lncRNAs in accordance with the data exported from starBase. Cytoscape 3.6 was applied in network analysis.

### 2.2. Patients

In total, 14 samples of normal tissue, 29 low-grade glioma samples, and 49 high-grade glioma samples were collected. These 14 normal tissue samples and 78 glioma samples were collected from the Department of Neurosurgery of Yijishan Hospital of Wannan Medical College. The 14 normal samples were extracted from damaged brain tissue of patients with brain trauma. The diagnosis of glioma was performed following the WHO criteria [1]. The inclusion criteria were as follows: Patients of 40-80 years of age with no serious chronic disease, no history of other cancers, and no history of exposure to pollutants (namely, radioactive pollution and carcinogenic chemical pollution). Glioma tissues were stored in liquid nitrogen.

### 2.3. Cell Culture and GSC Culture

The human glioma cell lines U251 and U87MG were provided by the Chinese Academy of Science Cell Bank (Shanghai, China). The cell lines were cultured in high glucose Dulbecco's modified Eagle medium (DMEM) containing 10% fetal bovine serum without penicillin and streptomycin. Cells were cultured at 37°C under 5% CO_2_.

To propagate U87MG and U251 glioblastoma stem-like cells (U87GS, U251GS), we used the same method as Si Liu et al. [11].

### 2.4. Transfection

ASB16-AS1 small interfering RNA (siRNA) and negative control (NC) siRNA (100 nM) were synthesized by Guangzhou RiboBio Co. Ltd. (Guangzhou, China). Transfection was performed with riboFECT™ CP (Guangzhou RiboBio Co., Ltd.) following the manufacturer's protocol. ASB16-AS1 siRNA-1 sequence was 5'-GGTTCTGAATCATTCAGTT-3' and ASB16-AS1 siRNA-2 sequence was 5'-AAGCATCTTCAGTTTTCATATGA-3'.

Total RNA was extracted 24 h after transfection for reverse transcription-quantitative polymerase chain reaction (RT-qPCR) analysis. Total protein was extracted 48 h after transfection for western blot analysis.

### 2.5. RT-qPCR

Total RNA was extracted from tissues or cells using TRIzol and stored at -80°C. LncRNA and messenger RNA (mRNA) were reverse-transcribed into complementary DNA with the RevertAid First Strand cDNA Synthesis Kit (#K1622; Thermo Fisher Scientific, Inc., Waltham, MA, USA) on a GT9612 Gradient Thermal Cycler. RT-qPCR was performed with the QuantiNova™ SYBR® Green PCR kit on a QuantStudio 3 Real-Time PCR System. GAPDH served as an internal standard.

The experimental protocol and reaction conditions complied with the manufacturer's instructions. The sequences of the primers are as follows: GAPDH forward primer, 5'-GCCTGCTTCACCACCTTCT-3', and GAPDH reverse primer, 5'-GAACGGGAAGCTCACTGG-3'; ASB16-AS1 forward primer, 5'-CGGCCCTGAGGCAAACATAC-3', and reverse primer, 5'-TGAAACACTGCGCCAACTTC-3'.

### 2.6. Real-Time Cellular Analysis (RTCA)

The present study monitored the rate of proliferation, migration and invasion of cells in real time with the xCELLigence RTCA System. Cellular impedance was recorded in real time using the xCELLigence RTCA System, and the cell index, which reflects the state of cell growth, was calculated based on cellular impedance. RTCA Station must be put in 37°C, 5% CO_2_ incubator 1 h before the start of the experiment. There were 3 groups for each cell line, namely, NC, ASB16-AS1 siRNA-1, and ASB16-AS1 siRNA-2, and each group was analyzed in 4 replicate wells. A Mann-Kendall trend test was performed to compare two RTCA curves (siRNA-1/siRNA-2 vs NC).

### 2.7. Proliferation Assay Using RTCA

E-Plate 16 was used for the proliferation assay. A total of 5,000 transfected cells in 150 *μ*l DMEM containing 10% serum were seeded in each well. Then, E-Plate was reinserted into RTCA Station. Schedule was run 20 min later.

### 2.8. Matrigel Invasion Assay Using RTCA

CIM Plate 16 was used for the Matrigel invasion assay. In total, 40 *μ*l diluted Matrigel (1:5 solutions; catalog no. 356234; BD Biosciences, Franklin Lakes, NJ, USA) was placed into each well of the upper chamber. The transfected cells were resuspended in serum-free medium, and a total of 40,000 cells in 100 *μ*l medium were added to each well of the upper chamber. Next, DMEM containing 10% serum was added to the lower chamber. The CIM plates were reinserted into the RTCA Station and run schedule.

### 2.9. Migration Assay Using RTCA

CIM Plate 16 was used for the migration assay. The transfected cells were resuspended in serum-free medium, and a total of 20,000 cells in 100 *μ*l medium were added to each well of the upper chamber. Next, DMEM containing 10% serum was added to the lower chamber. The CIM plates were reinserted into the RTCA Station and run schedule.

### 2.10. Western Blot Analysis

Following the corresponding treatments, proteins were extracted in accordance with the instructions of the Membranous and Cytoplasmic Protein Extraction Kit (Beyotime Institute of Biotechnology, Haimen, China). Antibodies against *β*-actin (13E5; rabbit monolonal antibody (mAb), 45 kDa), E-cadherin (24E10; rabbit mAb, 135 kDa), N-cadherin (D4R1H; rabbit mAb, 140 kDa), and vimentin (D21H3; rabbit mAb, 57 kDa) were provided by Cell Signaling Technology, Inc. (Danvers, MA, USA), and applied in western blotting. Immunodetection was performed with standard techniques.

### 2.11. Flow Cytometry

U87GS and U251GS cells transfected with ASB16-AS1 siRNA-1, siRNA-2, or NC were seeded into a 24-well plate at a density of 10^5^ cells/ml. To each well, 1 ml cell suspension was added. There were 3 groups (namely, NC, siRNA-1, and siRNA-2), and 3 wells were assessed per group. After incubation for 48 h at 37°C in the presence of 5% CO2, flow cytometry was used for cell cycle analysis.

### 2.12. Statistical Analysis

All data were expressed as the mean ± standard deviation. Statistical analysis was performed using R. Unpaired Student's* t*-test was performed to compare two groups. One-way analysis of variance (ANOVA) was used to compare multiple groups. Fisher's least significant difference test was applied for the comparison of means. Mann-Kendall trend test was performed to compare two RTCA curves. P<0.05 was considered to indicate a statistically significant difference.

## 3. Results

### 3.1. Predicted ceRNA Network of DElncRs

**Figure 1:**
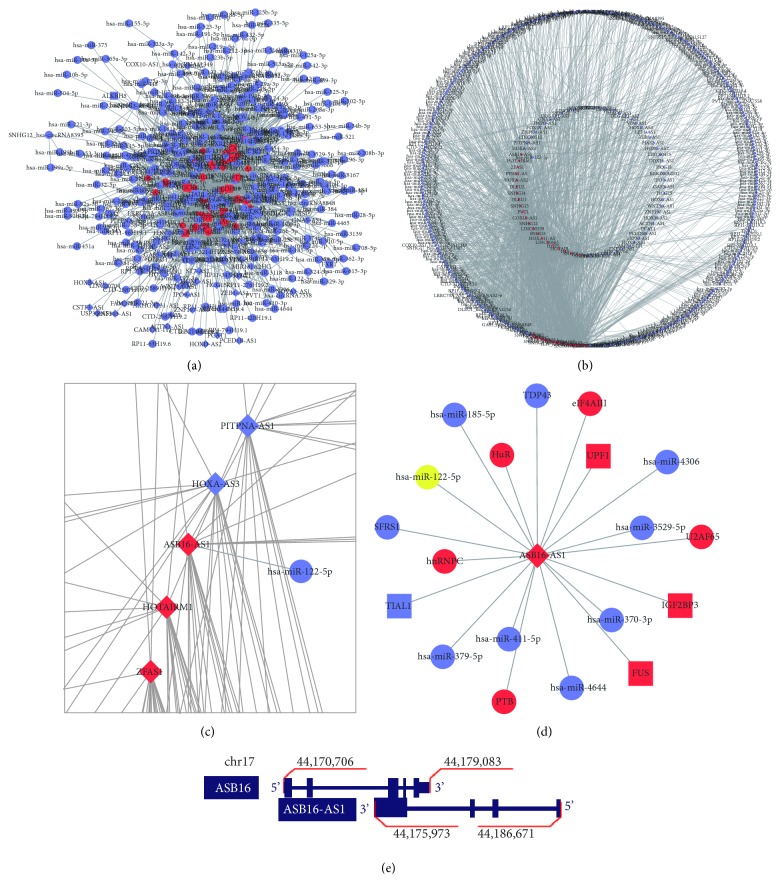
Bioinformatic analysis. The top 10% hub nodes are shown in red, while the remaining hub nodes are shown in blue. lncRNAs are represented as diamonds; genes with protein products are shown as rectangles; and other molecules are shown as circles. (a) A total of 178 lncRNAs within the differentially expressed genes met the condition of area under the curve >0.85. The ceRNA network for those 178 lncRNAs was constructed based on data exported from starBase. Not all lncRNA data could be identified in starBase, and only 72 lncRNAs were identified in the ceRNA network. (b) Degree sorted circle layout of graph (b), which contains two circular layouts. lncRNAs are introduced in the inner circular layout, while other RNAs are located in the outer circular layout. (c) Zoom in an area of (b) to show the details of ASB16-AS1 in network. (d) Nodes directly connected with ASB18-AS1 in the ceRNA network. (e) Localization of ASB16 and ASB16-AS1 on chromosome 17. The figure was constructed based on information from UCSC Genome Browser (genome.ucsc.edu).

For identification of DElncRs, the criteria used were |log_2_ fold-change| >1.0 and adjusted P<0.001. ROC analysis was conducted on each DElncR by R code. A total of 178 DElncRs were identified, of which AUC >0.85. The lncRNA-miRNA and lncRNA-protein interactions databases were downloaded from starBase. The predicted ceRNA network of those 178 DElncRs was built based on the data from starBase (Figures 1(a) and 1(b)). Not all the DElncRs could be identified in starBase, and only 72 lncRNAs were included in the ceRNA network. Top 10% hub genes were identified in line with the connectivity of nodes in network.

### 3.2. ASB16-AS1 Expression Levels Are Significantly Upregulated in Human Glioma Tissues and Correlate with WHO Grade

**Figure 2:**
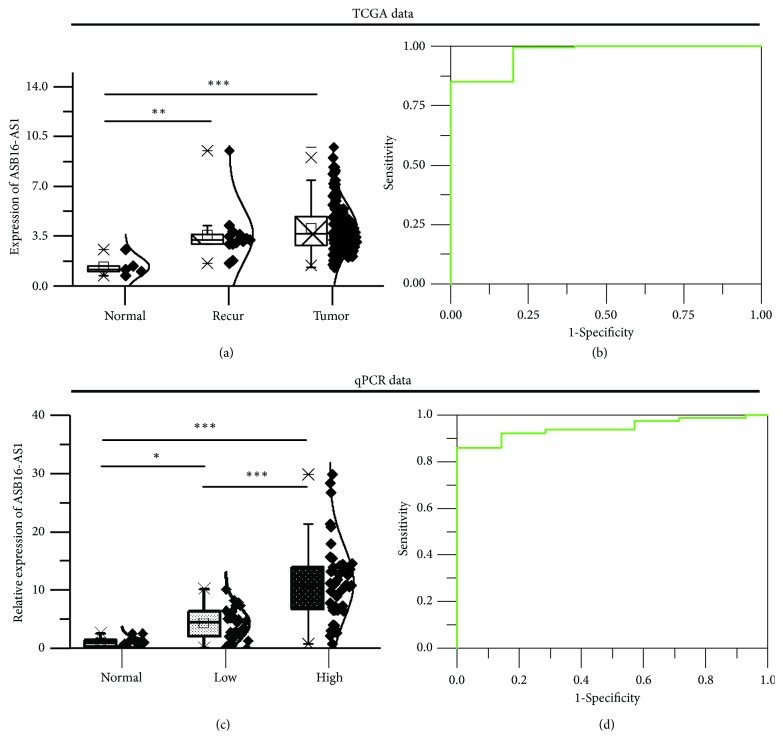
Expression of ASB16-AS1 in the TCGA database and in glioma tissues. (a) Boxplot based on TCGA FPKM data of ASB16-AS1. Data were evaluated by analysis of variance, and Fisher's LSD test was used for comparison of means. *∗*P<0.05, *∗∗*P<0.01, *∗∗∗*P<0.001. (b) ROC curve of expression of ASB16-AS1 in TCGA. AUC = 0.96923. (c) Boxplot based on the relative expression of ASB16-AS1, as detected by qPCR. High-grade gliomas exhibited significantly higher levels of ASB16-AS1 expression compared with low-grade gliomas. (d) ROC curve of qPCR data. AUC = 0.95065.

ASB16-AS1 has not been reported in other studies to date. Thus, the present study tried to verify if it has the value of continuing research. ASB16-AS1 exhibited a significant increase in tumors in the TCGA database compared with normal samples (Figure 2(a)), as well as a high sensitivity and specificity for diagnosing gliomas (Figure 2(b)). However, whether ASB16-AS1 is involved in glioma development remains unclear. RT-qPCR was used to detect the expression of ASB16-AS1 in human glioma tissues. The results indicated that the expression of ASB16-AS1 was obviously increased in 77 glioma tissues in comparison with 15 normal tissues. ASB16-AS1 expression in high-grade tumors (WHO grade III or IV) was further studied in comparison with low-grade tumors (WHO grade I or II). The results revealed that ASB16-AS1 expression was significantly correlated with WHO grade (Figures 2(c) and 2(d)). This suggests that high expression of ASB16-AS1 may be critical for tumor progression.

### 3.3. ASB16-AS1 Promotes Proliferation, Migration, and Invasion of Glioma Cells In Vitro

**Figure 3:**
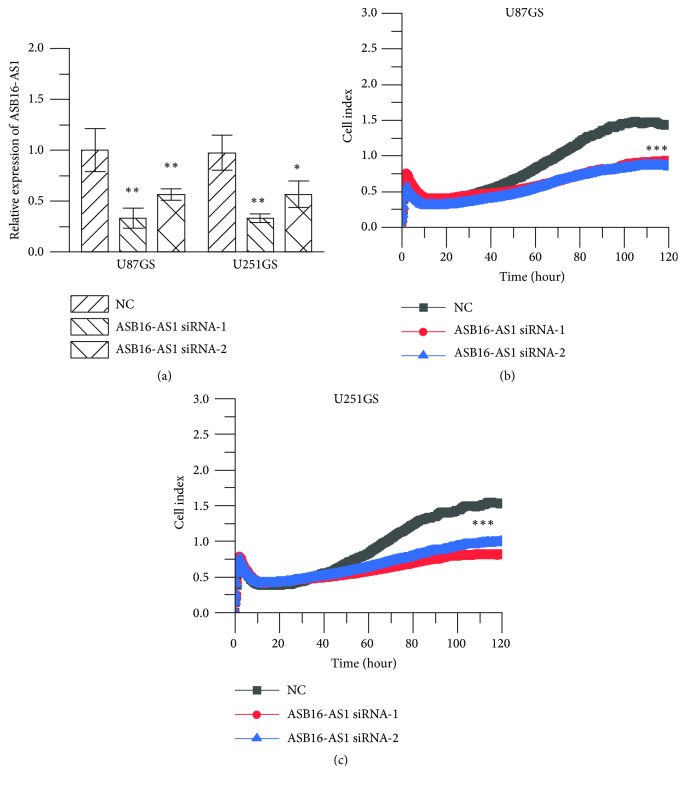
RTCA proliferation assays. (a) Inhibition efficiency was quantified using qPCR upon transfection of NC and ASB16-AS1 siRNA-1/siRNA-2. (b) Growth curve of U87GS transfected with NC, ASB16-AS1 siRNA1, and siRNA-2. (c) Growth curve of U251GS cells transfected with NC, ASB16-AS1 siRNA1, and siRNA-2.

**Figure 4:**
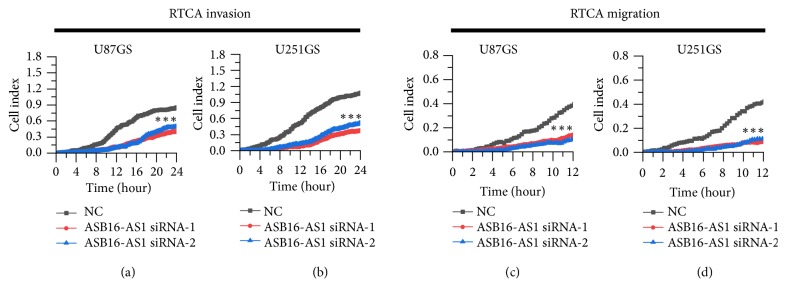
RTCA invasion/migration assays. (a and b) Results of RTCA invasion assays. (c and d) Results of RTCA migration assays. *∗∗∗*P<0.001.

To study the potential function of ASB16-AS1 in gliomas, the RTCA system was employed to determine whether ASB16-AS1 was involved in glioma cell proliferation, migration, and invasion. The results of RTCA proliferation assay suggested that the number of proliferating U87GS and U251GS cells transfected with ASB16-AS1 siRNA was reduced compared with the group transfected with NC (Figure 3). This observation suggests that ASB16-AS1 has a positive effect on cell proliferation. Next, RTCA invasion and migration assays were conducted to examine the role of ASB16-AS1 in glioma cells. It was observed that the proliferation, invasion, and migration abilities of the cells were significantly suppressed upon knocking down ASB16-AS1 in U87GS and U251GS cells (Figure 4).

### 3.4. ASB16-AS1 Activates the EMT Pathway

To assess whether the EMT signaling pathway could be impacted by ASB16-AS1, cells were transfected with NC or ASB16-AS1 siRNA, and key proteins of the EMT signaling pathway were detected by western blotting.

**Figure 5:**
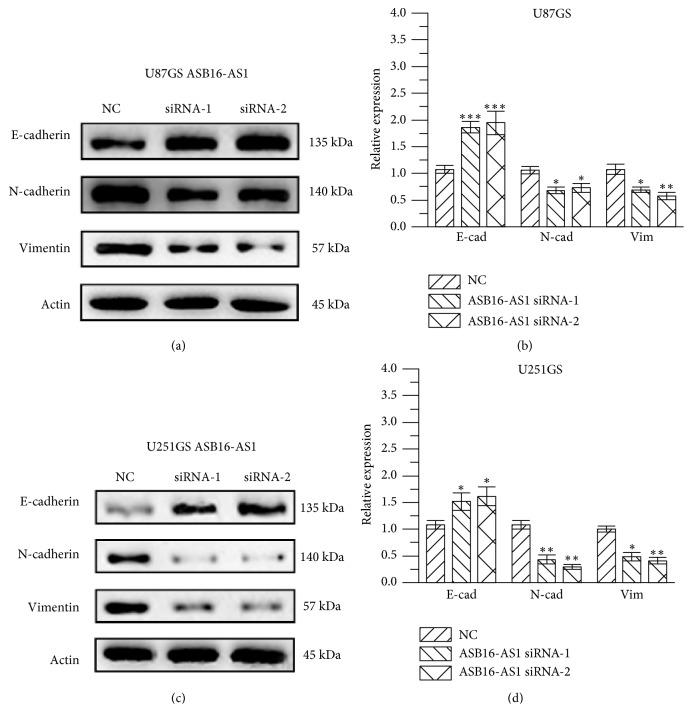
Expression of key proteins of the EMT signaling pathway in U87GS and U251GS cells. (a and b) Changes in key proteins of the EMT signaling pathway in U87GS cells upon knockdown of ASB16-AS1. In the siRNA-1/siRNA-2 groups, E-cadherin expression was significantly increased (P<0.001), while N-cadherin and vimentin expression was significantly decreased (both P<0.05). (c and d) Changes in key proteins of the EMT signaling pathway in U251GS cells upon knockdown of ASB16-AS1. In the siRNA group, E-cadherin expression was significantly increased (P<0.05), while N-cadherin and vimentin expression was significantly decreased (P<0.01 and P<0.05, respectively).

E-cadherin expression was significantly increased in the siRNA group compared with the NC group. The expression of vimentin and N-cadherin was decreased in the siRNA group compared with the NC group (Figure 5). Taken together, these results demonstrate that ASB16-AS1 is able to active the EMT signaling pathway in order to affect the development of tumors.

### 3.5. ASB16-AS1 siRNA Inhibits U251GS and U87GS Cell Cycle

**Figure 6:**
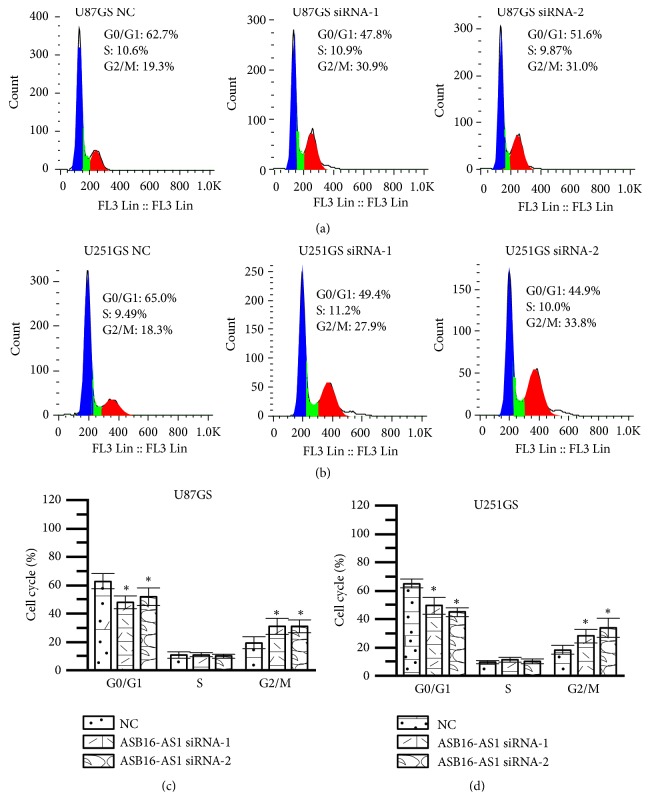
Cell cycle distributions at 48 h after transfection with NC, siRNA-1, or siRNA-2. (a) Flow cytometry results of U87GS cells. (b) Flow cytometry results of U251GS cells. (c) Statistical analysis results of U87GS cell cycle. (d) Statistical analysis results of U251GS cell cycle. *∗* P<0.05 compared with the NC group.

After 48 h of transfection, the cell cycle distribution was studied. The percentage of cells in G2/M phase in the ASB16-AS1 siRNA-1 group was 30.9±5.4% (U251GS) and 27.9±4.6% (U87GS), while such percentage in the ASB16-AS1 siRNA-2 group was 31.0±4.5% (U251GS) and 33.8±6.5% (U87GS). Compared with the NC groups, the percentage of cells in G2/M phase was significantly increased in the ASB16-AS1 siRNA groups (both P<0.05). This result revealed that the inhibition of the expression of ASB16-AS1 could affect the progression of the cell cycle and increase the number of cells in G2/M phase (Figure 6)

## 4. Discussion

ncRNA molecules are increasingly known to be involved in the regulation of various cellular activities, including chromatin structure [12], miRNA “sponge” [6, 13, 14], splicing [15], and translation [16]. Lu et al. [17] reported that lncRNA MIR100HG had two embedded miRNAs, miR-100 and miR-125b.

This study aimed to identify a lncRNA involved in the development of glioma. At present, there are numerous international databases that are publicly available for researchers. Previous studies on miRNAs have demonstrated that lncRNAs can regulate the functions of miRNAs via the ceRNA mechanism. Based on the ceRNA theory, an interaction network can be built. In the present study, TCGA glioblastoma RNAseq FPKM data were first downloaded to identify all known lncRNAs. Genes were annotated using data downloaded from HGNC, and differential analysis was performed. The filter criteria were set to |log_2_ fold-change| >1.0 and adjusted P<0.001. Since the HGNC database has a total of 3,861 known lncRNAs, setting the filter criteria too stringent would yield an insufficient number of selected lncRNAs, which would make the network analysis less useful. When assessing the results of a differential analysis, screening by P values is more effective than screening by fold-change. Accordingly, the threshold of |log_2_ fold-change| was set to 1 to increase the number of selected lncRNAs and to reduce the probability of potential lncRNAs being excluded. The adjusted P value threshold was set to 0.001 to improve the significance of the selected genes. In total, 471 lncRNAs that had |log_2_ fold-change| >1.0 and adjusted P < 0.001 were obtained. Since it is not sufficient to screen out lncRNAs by only differential analysis, ROC analysis was used. In addition, lncRNAs with AUC >0.85 were used to build the ceRNA network. A network with 417 nodes and 1,650 edges was built. Based on the connectivity of the nodes in the network, the top 10% hub genes were identified.

The differential expression of ASB16-AS1 in glioma tissues was first validated. ASB16-AS1 expression was significantly increased in tumor tissues and was significantly higher in high-grade glioma tissues than in low-grade glioma tissues. This finding agrees with the TCGA database. AUC > 0.9 suggests that ASB16-AS1 may serve an important role in tumor diagnosis.

To verify the function of ASB16-AS1, ASB16-AS1 was knocked down in U87GS and U251GS cells. Then, the proliferation, invasion, and migration abilities of these cells were detected with the RTCA system. As the results revealed, the function of glioma cells was suppressed by knocking down ASB16-AS1. In addition, compared with the NC group, in the siRNA group, the percentage of cells in the G2/M phase was increased, while that of cells in G0/G1 was decreased. In addition, the EMT signaling pathway was affected by knocking down ASB16-AS1. Generally, EMT induces rather suppression of cellular proliferation or has no effect on cellular proliferation, although it enhances the migratory or invasive ability of cells. In the present study, knockdown of ASB16-AS1 inhibited cell proliferation and suppressed EMT in glioma cells. Therefore, it is difficult to explain how ASB16-AS1 regulates the proliferation of glioma cells. Thus, the molecular mechanism of ASB16-AS1 remains unknown, and further studies should be conducted to unravel how ASB16-AS1 affects EMT, cell proliferation, and cell cycle.

ASB16-AS1 is transcribed from the antisense strand of protein-coding genes and it overlaps with exonic and intronic regions (Figure 1(e)). The protein encoded by the ASB16 gene belongs to the ankyrin repeat motif protein family and contains a suppressor of cytokine signaling (SOCS) box, which serves a role as a coupling reagent for SOCS proteins, as previously reported. Liu et al. identified SPSB1, a SOCS-box protein that contains a Spry domain, as the regulator of the TGF-*β* signaling pathway, which clearly correlates with human tumor progression [18]. Du et al. revealed that ASB3 may inhibit colorectal cancer metastasis by retarding the epithelial-mesenchymal transition [19]. Proteins of the ASB family may participate in the regulation of tumor-associated pathways. Since the antisense RNA strand of a certain gene holds complementary sequence compared with its corresponding mRNA, the expression level of ASB16-AS1 can possibly affect that of ASB16, or even other genes in the ASB family. Currently, few studies have been performed on the functions of the ASB16 protein, and whether this protein is associated with glioma progression requires further investigation. Therefore, further studies should be conducted in order to reveal whether ASB16-AS1 functions by affecting the ASB family.

## 5. Conclusions

Our results indicate that ASB16-AS1 regulates EMT pathway. As a tumor promoter in glioma cells, knowing down ASB16-AS1 inhibits glioma proliferation, invasion, and migration in vitro.

## Data Availability

The data used to support the findings of this study are included within the article.
